# Acquired resistance to venetoclax (ABT-199) in *t(14;18)* positive lymphoma cells

**DOI:** 10.18632/oncotarget.12132

**Published:** 2016-09-20

**Authors:** Juraj Bodo, Xiaoxian Zhao, Lisa Durkin, Andrew J. Souers, Darren C. Phillips, Mitchell R. Smith, Eric D. Hsi

**Affiliations:** ^1^ Department of Laboratory Medicine, Robert J. Tomsich Pathology and Laboratory Medicine Institute, Cleveland Clinic, Cleveland, OH, USA; ^2^ Oncology Discovery, AbbVie, Inc., Chicago, IL, USA; ^3^ Department of Hematology and Medical Oncology, Taussig Cancer Institute, Cleveland Clinic, Cleveland, OH, USA

**Keywords:** venetoclax, resistance, follicular lymphoma

## Abstract

The chromosomal translocation *t*(*14;18*) in follicular lymphoma (FL) is a primary oncogenic event resulting in BCL-2 over-expression. This study investigates activity of the BH3 mimetic venetoclax (ABT-199), which targets BCL-2, and mechanisms of acquired resistance in FL.

The sensitivity of FL cells to venetoclax treatment correlated with BCL-2/BIM ratio. Cells with similar expression of anti-apoptotic proteins, but with higher levels of BIM were more sensitive to the treatment. Venetoclax induced dissociation of BCL-2/BIM complex and a decrease in mitochondrial potential. Interestingly the population of cells that survived venetoclax treatment showed increased p-ERK1/2 and p-BIM (S69), as well as a decrease in total BIM levels. Venetoclax resistant cells initially showed elevated levels of p-AKT and p-Foxo1/3a, a dissociation of BIM/BCL-2/BECLIN1 complex, and a decrease in SQSTM1/p62 level (indicating increased autophagy) together with a slight decline in BIM expression. After stable resistant cell lines were established, a significant reduction of BCL-2 levels and almost total absence of BIM was observed.

The acquisition of these resistance phenotypes could be prevented via selective ERK/AKT inhibition or anti-CD20 antibody treatment, thus highlighting possible combination therapies for FL patients.

## INTRODUCTION

Follicular lymphoma (FL) is the most common indolent B-cell lymphoma in the United States [1]. FL is characterized by translocation *t(14;18)(q32;q12)*, which deregulates BCL-2 expression and increases the apoptotic threshold of the FL cells [2].

The imbalance in expression of pro- and anti-apoptotic proteins of BCL-2 family has been implicated in the development of various tumor types and resistance to chemotherapy [3]. This may often be the result of high-level expression of anti-apoptotic proteins that prevent cell death by sequestering BH3-only pro-apoptotic proteins, such as BIM, PUMA, and NOXA, and restrict activation of apoptosis. In such instances, up-regulation and binding of anti-apoptotic proteins to activator proteins favors cell survival [4].

ABT-737 and its orally active analog navitoclax (ABT-263) are BH3 mimetics that have been tested clinically in single agent and combination trials [5]. Venetoclax (ABT-199) is a second generation BCL-2 family inhibitor designed to have much less effect on BCL-XL and therefore minimal treatment-associated thrombocytopenia, which was a dose limiting toxicity in early trials with navitoclax [6]. Clinical trials with venetoclax, alone and in combination, are proceeding in CLL and lymphomas [7]. The *t(14;18)* translocation in FL makes this malignancy a rational target for BH3 mimetics. While venetoclax has shown single agent activity in patients with FL, the overall response rates have not been as high as patients with CLL. An understanding of the *in vitro* and *in vivo* activity of venetoclax in FL could therefore assist clinical development by identifying potential predictive biomarkers, mechanisms of resistance, and rationale for therapeutic combinations that might prevent or overcome resistance. Research in this field has been impeded by lack of representative FL cell lines and appropriate animal models. In this study, we utilized patient samples and *t(14;18)* positive cell lines derived from a patient with leukemic phase FL, WSU-FSCCL [8] and from a patient with transformed FL, FC-TxFL2 [9]. We also developed venetoclax-resistant cell lines by continuous treatment with venetoclax to investigate mechanisms of resistance.

## RESULTS

### Induction of apoptosis in primary FL cells after venetoclax treatment

**Figure 1:**
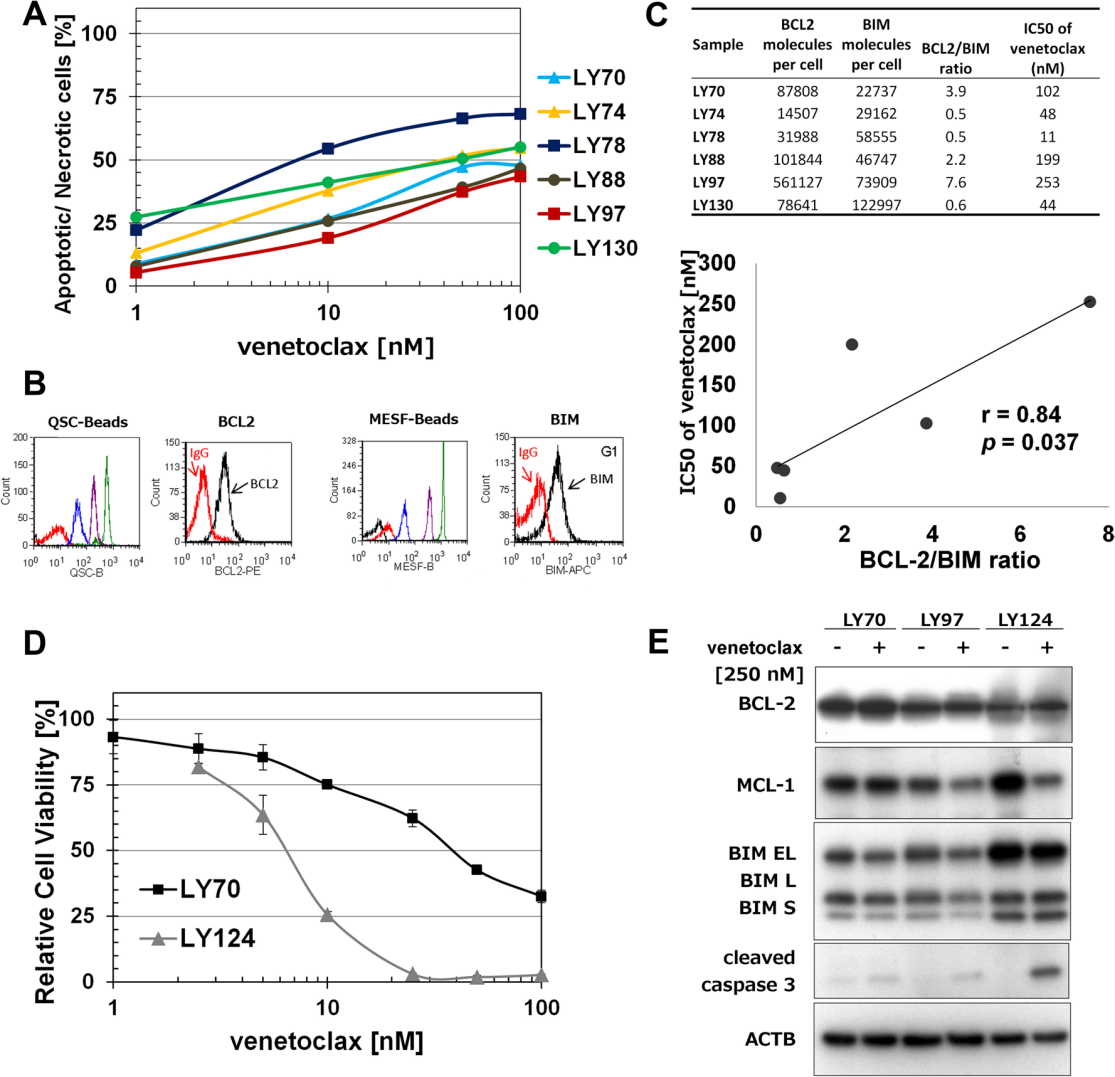
Venetoclax induces proliferation inhibition and apoptosis in *t(14;18)* positive cells (**A**) Apoptosis induction in primary FL samples after venetoclax treatment. Primary cells were treated with venetoclax for 4 H and Annexin-V/7-AAD based flow cytometry assay was performed to determine the percentage of apoptotic/necrotic cells. (**B**) An example (sample LY74) of quantitative flow cytometry analysis of BCL-2 and BIM expression (**C**) Correlation between BCL-2/BIM ratio and IC_50_ values of venetoclax. BCL-2 and BIM expression (molecule number/cell) was analyzed by quantitative flow cytometry assay. IC_50_ of venetoclax was calculated using data collected in 1a. (**D**) Cytotoxicity of venetoclax in primary FL samples treated for 72 H and analyzed with WST-1 assay. (**E**) A comparison of BCL-2, MCL-1, BIM, and cleaved caspase-3 protein expressions in primary FL samples.

Venetoclax treatment induced a concentration – dependent decrease in cell viability in six FL primary samples (Figure 1A). The LY78 sample was the most sensitive (IC_50_ = 11 nM) and the LY97 sample the most resistant (IC_50_ > 200 nM) to venetoclax treatment. To inform upon the range of venetoclax responses observed, we determined the expression of BCL-2 and BIM in primary FL samples by flow cytometry [10] (Figure 1B). Subsequent flow cytometric analysis of BCL-2 and BIM levels revealed a significant (*p < 0.05*) positive correlation between BCL-2/BIM ratio and IC_50_ values of venetoclax in tested FL samples (Figure 1C). Similarly, FL samples that had lower levels of BIM protein and comparable level of BCL-2 protein analyzed by WB were more resistant to venetoclax treatment (Figure 1D, 1E).

### Venetoclax inhibits proliferation and induces apoptosis in FL cell lines

**Figure 2:**
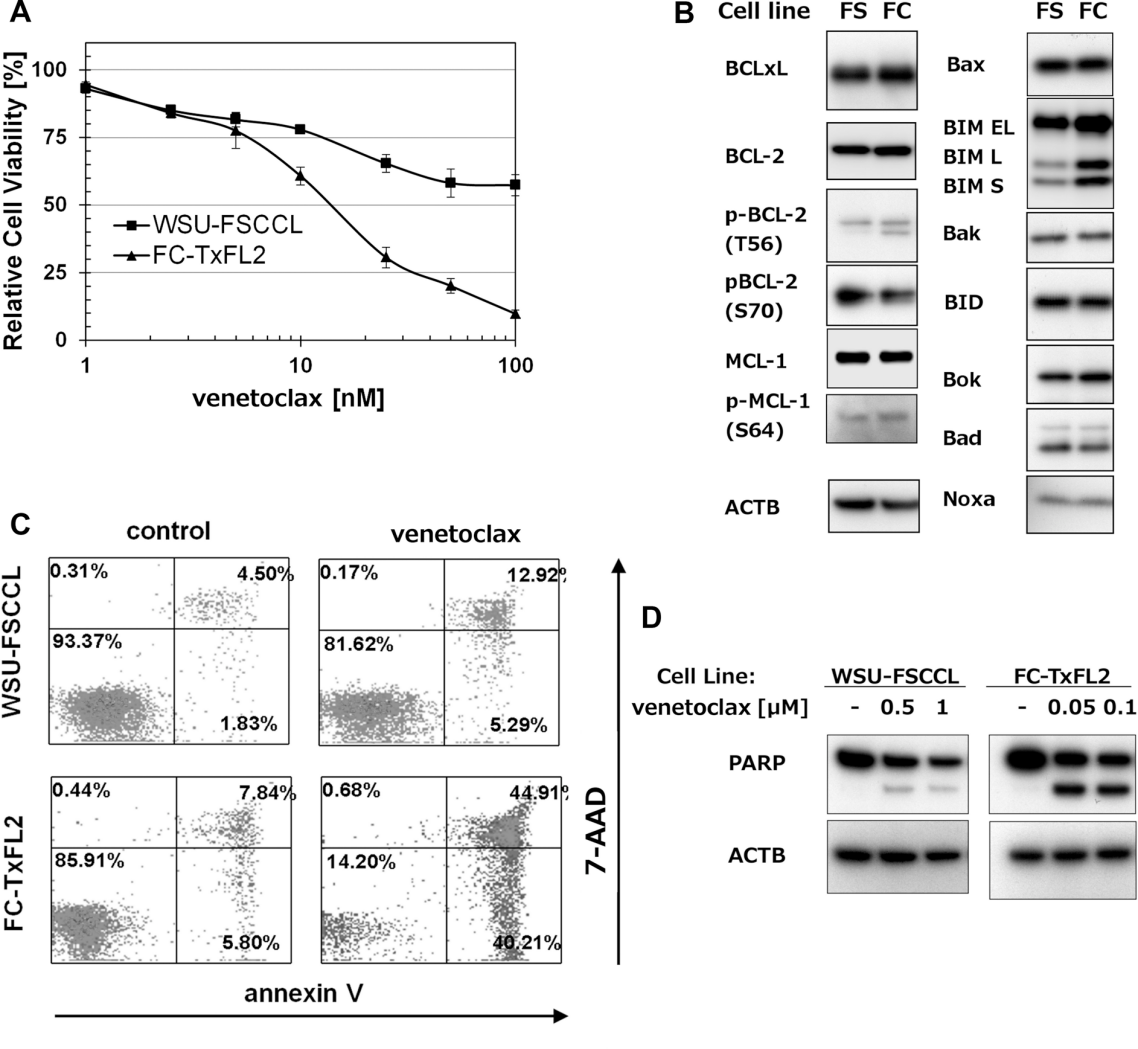
The effect of venetoclax on *t(14;18)* positive cell lines (**A**) Cytotoxicity of venetoclax in FL cell lines treated for 72 H and analyzed with WST-1 assay. (**B**) A comparison of pro- and anti-apoptotic proteins expression in untreated WSU-FSCCL (FS) and FC-TxFL2 (FC) cell lines. (**C**) Annexin-V/7-AAD analysis of FL cell lines treated with 100 nM venetoclax for 24 H. (**D**) WB analysis of cleaved PARP in FL cell lines after 24 H venetoclax treatment.

The effect of venetoclax was further tested in two *t(14;18)* positive cell lines, WSU-FSCCL and FC-TxFL2. FC-TxFL2 cells (IC_50_ = 7 nM) were more sensitive to venetoclax treatment than WSU-FSCCL cells (IC_50_ = 110 nM) (Figure 2A). WB analysis showed similar levels of anti-apoptotic proteins, such as BCL-XL, BCL-2 and MCL-1 in both WSU-FSCCL (FS) and FC-TxFL2 (FC) cell lines (Figure 2B). Likewise, the levels of tested pro-apoptotic proteins, such as BAX, BID, BOK, BAD and NOXA, were comparable. The only exception was BIM protein. Levels of isoforms BIM EL, L, and S were significantly higher in FC-TxFL2 cell line than in WSU-FSCCL. Analysis of apoptosis induction using Annexin V/7-AAD assay (Figure 2C) and analysis of cleaved PARP (Figure 2D) confirmed higher sensitivity of FC-TxFL2 cells to the venetoclax treatment in comparison to WSU-FSCCL cells. This further suggested that FL cells with a relatively low BCL-2/BIM ratio are more sensitive to venetoclax treatment than the cells with low BIM and high BCL-2 levels.

### Disruption of BCL-2/BIM complex and activation of caspase-dependent apoptosis

**Figure 3:**
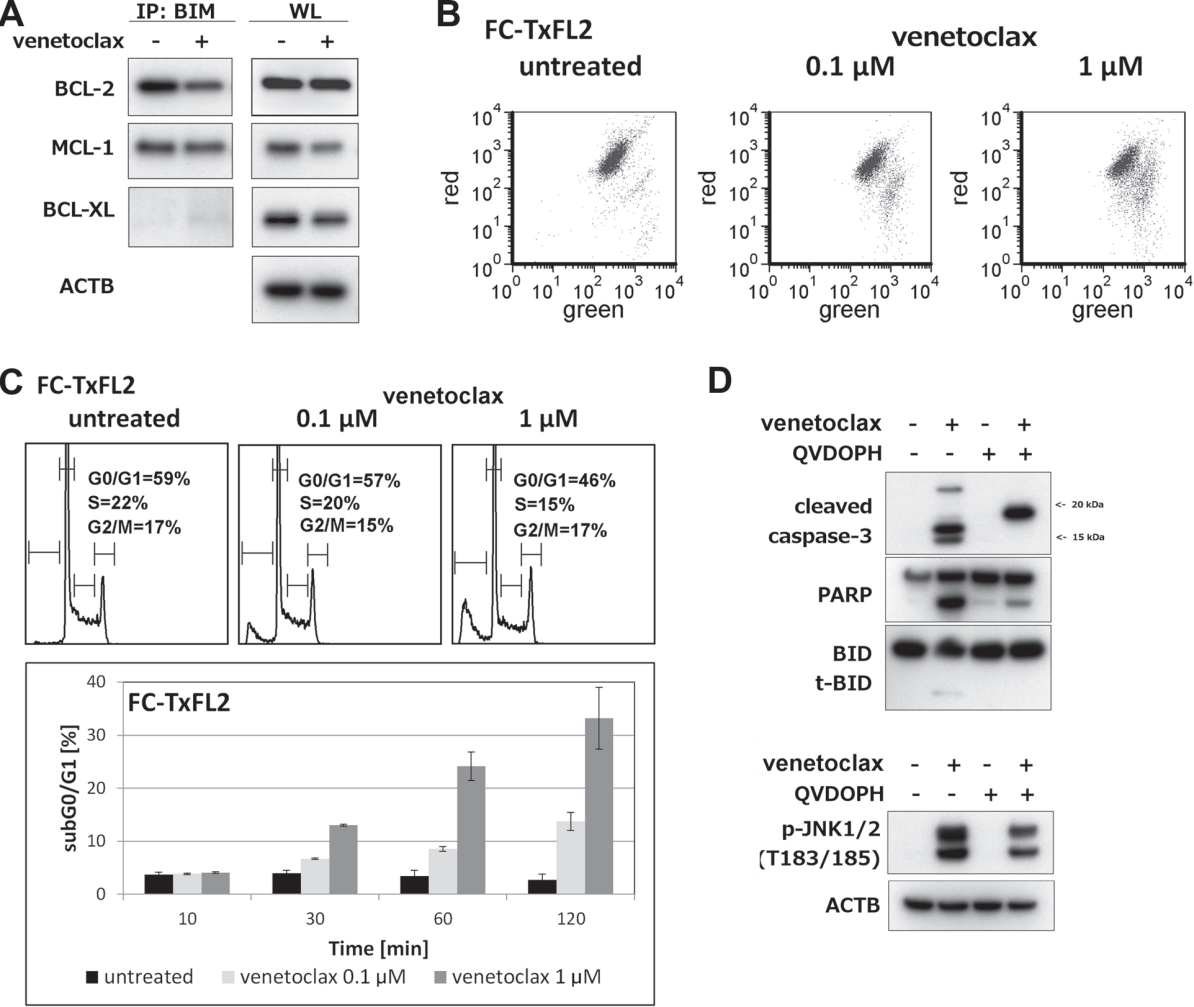
Cellular events proceeding and accompanying venetoclax induced apoptosis in FC-TxFL2 cell line (**A**) BIM protein immunoprecipitation followed by BCL-2, MCL-1 and BCL-XL WB detection of lysates of FC-TxFL2 cells treated with 100 nM venetoclax for 2 H. (WL – whole cell lysate) (**B**) Decrease of mitochondrial potential after 1 H venetoclax treatment analyzed by JC-1 assay. (**C**) Cell cycle analysis cells treated with venetoclax for 2 H and analysis of subG0/G1 apoptotic cells treated with venetoclax for 10, 30, 60 and 120 minutes. (**D**) Inhibition of caspase-3 activation, PARP and BID cleavage (t-BID) and decrease of JNK1/2 phosphorylation with pan caspase inhibitor Q-VD-OPH after 2 H venetoclax treatment.

To further study the role of BIM protein in venetoclax-induced apoptosis, immunoprecipitation (IP-WB) using BIM antibody was used. IP-WB showed a decrease in BCL-2/BIM complex levels in venetoclax-treated FC-TxFL2 cells (Figure 3A). Levels of MCL-1/BIM remained the same, while a slight increase of BCL-XL in complex with BIM was detected. Moreover, a rapid decrease in the mitochondrial membrane potential was observed (Figure 3B). Venetoclax treatment modified the cell cycle, inducing a decrease in G0/G1 and S-phase along with an increase in sub-G0/G1 apoptotic cells (Figure 3C). The treatment also induced an activation of caspase-3, JNK1/2 and a cleavage of BID protein. However, an inhibition of caspase activation decreased JNK1/2 phosphorylation and eliminated BID cleavage showing that these events were the result of active apoptosis (Figure 3D). In conclusion, venetoclax induced a release of BIM protein from BCL-2 that associated with activation of the intrinsic apoptotic pathway.

### Activation of ERK1/2 protects cells against venetoclax-induced apoptosis

**Figure 4:**
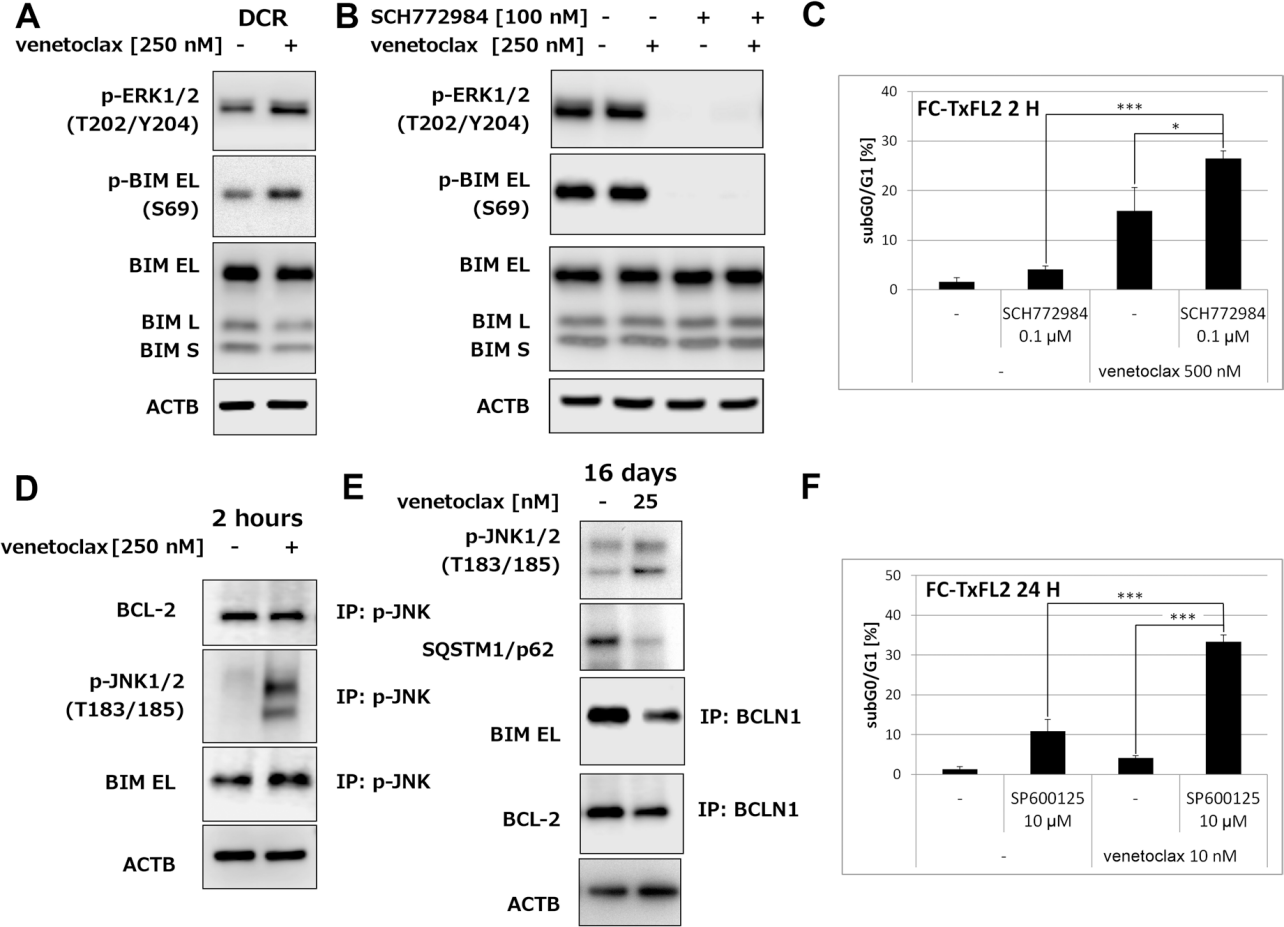
Activation of ERK1/2 and JNK1/2 in FC-TxFL2 cells treated with venetoclax (**A**) Expression of p-ERK1/2, p-BIM and BIM in viable FC-TxFL2 cells isolated using dead cell removal kit (DCR) after 2 H venetoclax treatment. (**B**) Inhibition of ERK1/2 and p-BIM using ERK inhibitor SCH772984 in whole cell population after 2 H treatment. (**C**) Potentiation of venetoclax – induced apoptosis analyzed as subG0/G1 cell population by ERK inhibitor (*p < 0.05, ***p < 0.001). Cells were exposed to SCH772984 for 24 H followed by 2 H treatment with venetoclax. (**D**) Detection of BCL-2 and BIM proteins after immunoprecipitation with p-JNK1/2 antibody after 2 H venetoclax treatment. (**E**) Detection of autophagy using SQSTM1/p62 marker and Beclin1 (BCLN1) immunoprecipitation. Cells were treated with the increasing concentrations of venetoclax (up to 25 nM) for 16 days. (**F**) Analysis of apoptosis induction in FC-TxFL2 cells after SP600125/venetoclax treatment for 24 H (***p < 0.001).

Interestingly, an analysis of ERK1/2 activation in cells surviving venetoclax treatment (dead cells were removed using Dead Cell Removal kit (DCR)) showed an increase of phospho-ERK1/2 together with an increase of BIM (S69) phosphorylation and slight decrease of total BIM protein (Figure 4A). Treatment with specific ERK inhibitor SCH772984 completely inhibited both phospho-ERK1/2 and phospho-BIM (S69) (Figure 4B). The level of total BIM protein remained unaffected. Detection subG0/G1 population as a marker of apoptosis revealed significant (*p < 0.05*) potentiation of venetoclax induced apoptosis using ERK inhibition in FC-TxFL2 cells (Figure 4C), but not in WSU-FSCCL cells (data not shown). Thus, ERK pathway plays a significant role in the initiation of acquired resistance of FC-TxFL2 cells to venetoclax in a transient manner, but ERK pathway activation is not required for maintenance of venetoclax acquired resistance.

### Increased activity of JNK1/2 and autophagy in cells after a long term venetoclax treatment

IP-WB using p-JNK1/2 antibody showed an increase in BIM EL/p-JNK1/2 levels with no effect on BCL-2/p-JNK1/2 complex (Figure 4D) after 2 hours of venetoclax treatment. Continuously increasing concentrations (1 − 25 nM) of venetoclax for 16 days kept JNK1/2 activated and almost completely eliminated SQSTM1/p62, which is consistent an increase of autophagy [11] in FC-TxFL2 cells (Figure 4E). Moreover, IP-WB showed a release of Beclin1 (BCLN1) from BIM EL/BCLN1 and BCL-2/BCLN1 complexes after the treatment. A non-specific JNK inhibitor SP600125 significantly (*p* < 0.001) enhanced the apoptotic effect of venetoclax (Figure 4F).

### Inhibition of PI3k potentiates venetoclax-induced apoptosis

**Figure 5:**
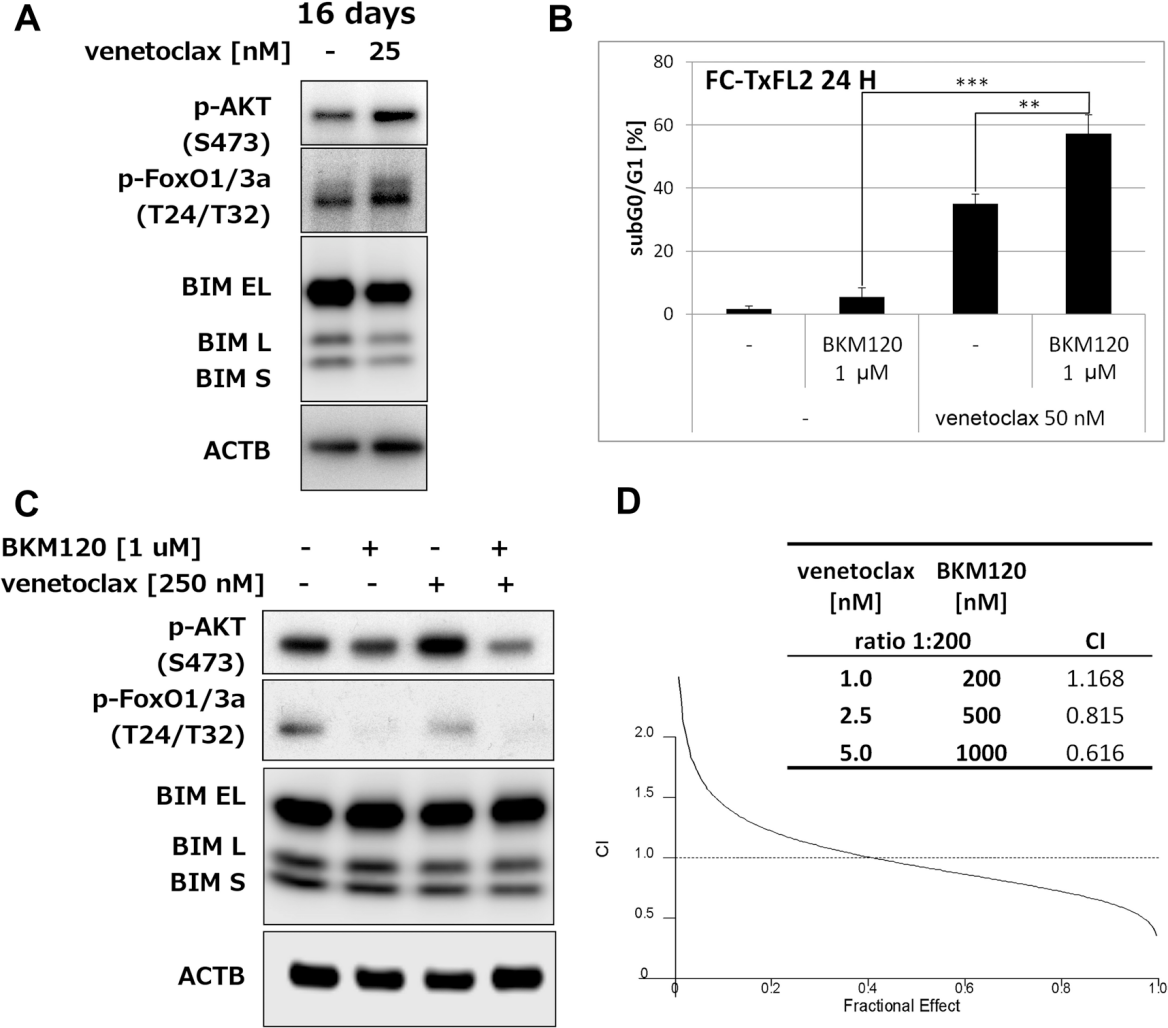
AKT pathway inhibition potentiates venetoclax induced apoptosis and proliferation inhibition (**A**) Western Blot analysis of p-AKT, p-Foxo1/3a and BIM in cells after 16 days' cultivation in the presence of increasing concentrations of venetoclax (up to 25 nM). (**B**) BKM120 potentiates venetoclax-induced apoptosis in FC-TxFL2 cells (** *p<0.01*, *** *p<0.001*). (**C**) Analysis of p-AKT, p-Foxo1/3a and BIM protein levels in cells treated with pan-PI3k inhibitor BKM120/venetoclax combination for 2 H. (**D**) Analysis of combination effect on proliferation inhibition using WST-1 assay in FC-TxFL2 cells after 72 H BKM120 (0.2 − 1 μM)/venetoclax (1 − 5 nM) (constant ratio 200:1) treatment. Combination index (CI) values were used to determine the combined effect as synergistic (<1), additive (=1), or antagonistic (>1) using Calcusyn software.

The 16 days treatment also increased levels of phosphorylated AKT (S473) and Foxo1/3a (T24/T32), while it decreased total BIM levels (Figure 5A). A combination of pan-PI3k inhibitor BKM120 with venetoclax significantly (*p < 0.01*) potentiated induction of apoptosis in comparison to either BKM120 or venetoclax single agent treatments (Figure 5B). BKM120 alone slightly decreased phospho-AKT and almost completely eliminated phosphorylated Foxo1/3a (Figure 5C) in FC-TxFL2 cells. The levels of BIM isoforms remained the same. BKM120 pretreatment followed by venetoclax further decreased p-AKT levels. Analysis of combined effect showed that BKM120/venetoclax treatment synergistically potentiated inhibition of proliferation of FC-TxFL2 cells (Figure 5D). This suggested that AKT pathway also plays an important role in protecting cells against venetoclax-induced apoptosis.

### Activity of venetoclax on FC-TxFL2 cells *in vivo*

**Figure 6:**
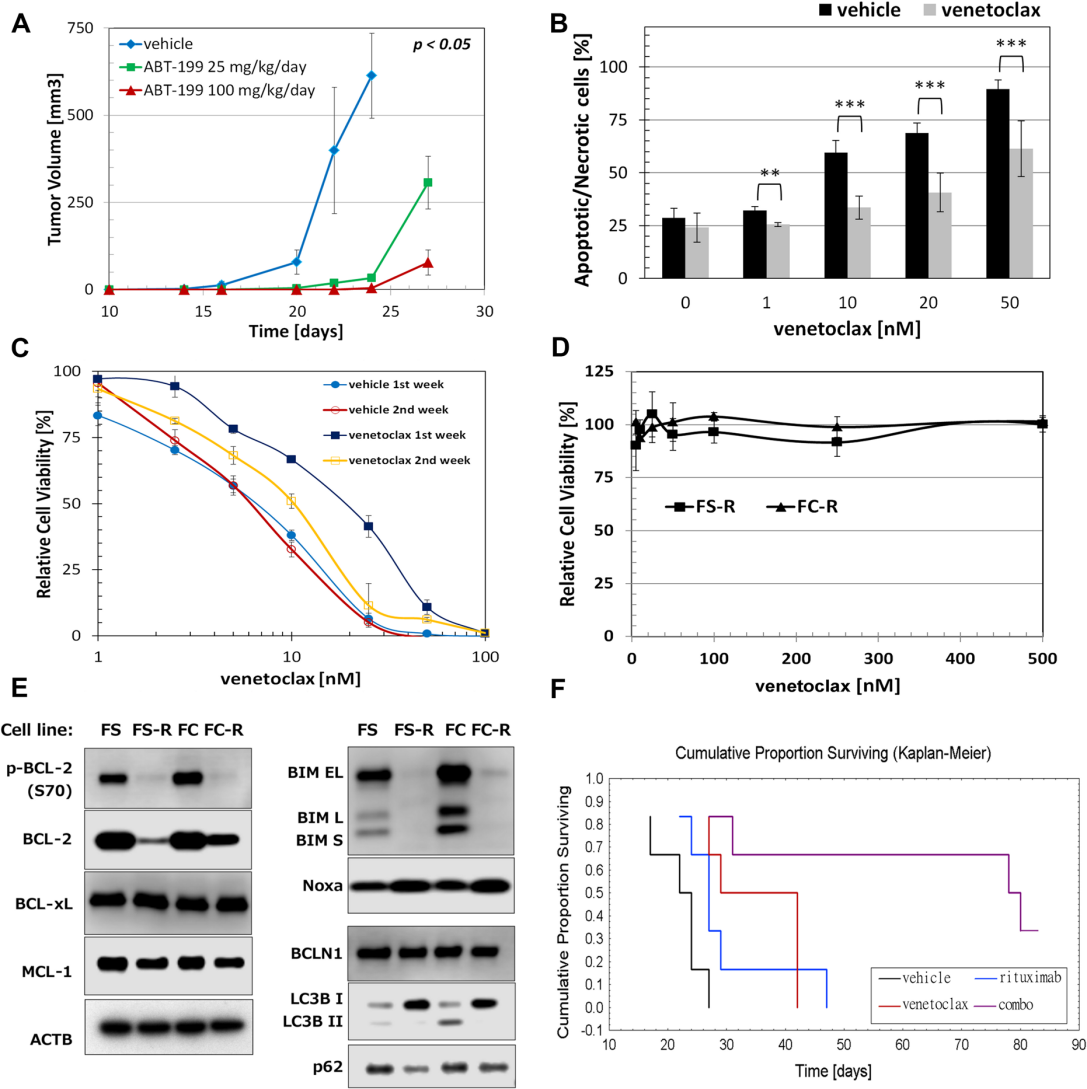
Acquired resistance to venetoclax in FL cells (**A**) Venetoclax delays tumor growth of FC-TxFL2 xenograft *in vivo*. Venetoclax was administered at the indicated doses once daily by gavage starting 5 days after the cell injection for 21 days. When tumors reached the maximum allowed size or at day 27, they were frozen or disintegrated for further experiments. Time refers to days from the injection of cells. (**B**) Increased resistance to venetoclax of FC-TxFL2 cells isolated from tumors previously treated with 50 mg/kg/day venetoclax in comparison to cells treated with vehicle (**p < 0.01, ***p < 0.001). Mice were treated until tumors reached maximum allowed size. Then the tumors were harvested, dissociated and the effect of 20 H venetoclax treatment on isolated cells *in vitro* was determined by flow cytometric analysis of annexin-v/7-AAD staining. (**C**) Comparison of sensitivity of FC-TxFL2 cells isolated from tumors treated with 100 mg/kg/day venetoclax to cells treated with vehicle one or two weeks after the isolation. Cells were treated with venetoclax for 72 H and analyzed by WST-1 assay. (**D**) Effect of venetoclax on viability of FS-R (WSU-FSCCL) and FC-R (FC-TxFL2) cells with acquired resistance to venetoclax. (**E**) Comparison of proteins regulating apoptosis and autophagy in original FL cell lines with cell lines with acquired venetoclax resistance. (**F**) Cumulative proportion surviving curve of NSG mice after combination venetoclax/rituximab treatment (*p < 0.05*). Mice were treated with vehicle, 8 mg/kg rituximab i.p. (only one dose) at day 14 and/or then daily with 50 mg/kg venetoclax by gavage. Venetoclax treatment continued until the tumor reached maximum allowed size. Survival curves were calculated when tumor size reached 1000 mm^3^. Six mice per group per experiment were used.

Oral administration of venetoclax (25 mg/kg/day or 100 mg/kg/day) significantly inhibited growth of FC-TxFL2 xenograft tumors in NSG mice (Figure 6A). However, after approximately 10 days, the tumors recovered and began to rapidly grow. When cells isolated from tumors treated *in vivo* were then treated with venetoclax *ex vivo*, they showed increased resistance to the venetoclax in comparison to the cells isolated from the tumors treated with vehicle only (*p < 0.01*) (Figure 6B). Interestingly, extending the culture of the venetoclax-treated FC-TxFL2 tumors for an additional week *ex vivo* in the absence of venetoclax rapidly reduced their resistance to venetoclax treatment *in vitro* (Figure 6C).

### Cells with acquired venetoclax resistance

To inform on the outgrowth of venetoclax treated FC-TxFL2 tumors *in vivo*, acquired resistant cell lines FS-R and FC-R were generated from the parental WSU-FSCCL and FC-TxFL2 cell lines by chronic exposure to increasing concentrations of venetoclax *in vitro* for more than two months. After the resistant cell lines were established, they were treated weekly with 500 nM venetoclax. This treatment had no effect on cell viability on either FS-R or FC-R cell line (Figure 6D). WB analysis revealed that resistant cells had down-regulated BCL-2 protein with almost undetectable levels of phospho-BCL-2 (S70) (Figure 6E). De-phosphorylation of BCL-2 (S70) may further decrease its anti-apoptotic activity [12, 13]. Interestingly, almost no changes were detected in levels of either MCL-1 or BCL-XL protein. Furthermore, both resistant cells had markedly decreased the expression of BIM protein. Besides BCL-2 and BIM, changes in LC3B I/II and SQSTM1/p62 levels were detected. While there was clear accumulation of LC3B I and complete removal of LC3B II protein in both resistant cell lines, there was a decrease of SQSTM1/p62 level that was especially evident in FS-R cells.

We have previously demonstrated potentiation between BCL-2 antisense oligonucleotides and a monoclonal antibody targeting CD20(rituximab) in mouse models [14], a combination with clinical relevance [15]. It also has been reported that rituximab inhibits both ERK 1/2 and AKT activation [16]. Subsequent co-treatment of FC-Tx-FL2 xenografts with venetoclax and rituximab enhanced mouse survival when compared to treatment with either agent alone (Figure 6F) and prevented the rapid establishment of venetoclax acquired resistance. Median survival values for vehicle, rituximab, venetoclax and combination treatment were 23, 27, 36 and 79 days, respectively.

## DISCUSSION

Despite BCL-2 overexpression in FL, early phase clinical trials showed less dramatic activity of venetoclax as a single agent in FL than in CLL patients [17]. We have also observed differential single agent sensitivity to venetoclax in a panel of primary FL samples, and found this sensitivity to correlate with BCL-2/BIM ratio. Likewise when two *t(14;18)* positive cell lines WSU-FSCCL and FC-TxFL2 with similar levels of BCL-2 were analyzed, a comparable correlation between the sensitivity to venetoclax and BIM levels was observed. Moreover, these cells had the same levels of anti-apoptotic protein such as BCL-XL and MCL-1, and similar levels of pro-apoptotic proteins such as BAX, BAK, BID, BOK, BAD and NOXA. These data corroborate earlier work in myeloma cell lines and murine lymphoid subsets where *BIM* gene silencing or *BIM* deletion reduced the activity of ABT-737 or venetoclax, respectively [18, 19]. Venetoclax mediated the dissociation of BCL-2/BIM complexes, leading to caspase-dependent apoptosis in *t(14;18)* positive FL cells that was associated with activation of JNK, ERK and AKT pathways

Correspondingly in the FC-TxFL2 xenograft model of FL, venetoclax significantly delayed tumor growth. This effect was dose-dependent, but associated with re-growth that only afforded an approximate 10 day increase in overall survival that may reflect the lower activity observed clinically with venetoclax in FL [17]. When cells isolated from FC-TxFL2 tumors were treated with venetoclax, increased resistance was observed in cells previously treated *in vivo* with venetoclax for two weeks in comparison to cells treated with vehicle only. This experiment indicated that FL cells can acquire resistance to venetoclax in a relatively short time. However, when cells were kept in medium without venetoclax for additional two weeks the resistance dissipated, suggesting that treatment holidays may re-sensitize FL cells.

To uncover evidence of cellular adaption to acute venetoclax treatment *in vitro*, we performed western blot analysis on proteins isolated from viable, non-apoptotic FC-TxFL2 cells after treatment with venetoclax. These cells showed higher levels of active ERK1/2, phosphorylated BIM (S69) and lower levels of total BIM when compared to the vehicle-treated FC-TXFL2 cells. It is known that when BIM is phosphorylated on serine 69, it is rapidly degraded via the proteasome pathway [20]. Together this data indicates a potential role of the ERK pathway in reducing the rate of the venetoclax-induced apoptosis in FC-TxFL2 cells. Indeed when cells were pretreated with ERK inhibitor SCH772984, a significant potentiation of venetoclax-induced apoptosis was detected. Similar potentiation of apoptosis induced by BCL-2 inhibition with ERK pathway inhibitor sorafinib was published previously [21]. Long term/low concentration venetoclax treatment induced activation of both JNK1/2 and AKT pathways in FC-TxFL2 cells as evidenced by an increase in phosphorylation status. Inhibition of JNK1/2 pathway with SP600125 [22] or the AKT pathway [23] with the PI3K inhibitor BKM120 [24] sensitized FC-TxFL2 cells to venetoclax. Although our initial thought was that JNK1/2 activation was mostly induced by apoptosis, with additional data it became evident that this pathway plays an important role in the apoptosis/autophagy balance. First, a moderate increase of p-JNK1/2/BIM levels suggesting an inhibition of the apoptotic function of BIM was detected. Second, together with JNK1/2 phosphorylation, events pointing to autophagy activation such as BIM/BECLIN1 and BCL2/BECLIN1 dissociation and SQSTM1/p62 degradation were observed. A detailed investigation of autophagy activation after venetoclax treatment is beyond the scope of this study; however, previous studies using ABT-737 [25–27] suggest that autophagy may play a significant role in cell adaption to BH3 mimetics. The importance of the AKT pathway in acquired resistance has also been published recently [23]. AKT can phosphorylate Foxo3a protein, a transcription factor of BIM expression, and negatively regulate BIM expression [28, 29]. When the AKT pathway was inhibited by the PI3-kinase inhibitor BKM120 [24] in this study, a significant decrease of phosphorylated Foxo1/3a proteins was detected. Although BKM120 pretreatment did not increase total BIM, it significantly potentiated the effect of venetoclax on apoptosis induction.

To further study the acquired resistance, both tested FL cell lines were incubated with increasing concentrations of venetoclax. After the resistant cell lines were established, venetoclax treatment had no significant effect on viability of the cells. Western blot analysis revealed decreased levels of BCL-2 and BIM proteins. With the lower levels of BCL-2, but especially with very limited levels of BIM protein, venetoclax lost the crucial target and activator required to induced apoptosis in these cells.

To augment the effect of venetoclax and prevent a rapid re-growth *in vivo*, co-treatment with rituximab, a reported inhibitor of ERK1/2 and AKT pathways [16] was examined. This combination treatment resulted in a substantial survival benefit over single agent venetoclax or rituximab treatment supporting clinical evaluation of this combination in FL.

Collectively, we have utilized a variety of *in vitro*, *ex vivo* and *in vivo* models to demonstrate that venetoclax is active in FL and this is dependent upon the ratio of BCL-2/BIM expression. However, the durability of response is limited due to the establishment of acquired resistance that is characterized by a loss in BCL-2 and BIM expression. Mechanistic studies revealed that JNK, AKT and ERK1/2 are activated in response to venetoclax treatment, which contributes to an acquired resistance phenotype. Concomitant pharmacological inhibition of JNK, AKT and ERK1/2 signaling pathways enhances venetoclax activity *in vitro* which helps to prevent establishment of acquired resistance. Importantly, these data inform in part on the mechanism by which rituximab in combination with venetoclax augments the survival of the FC-TxFL2 xenograft model of FL and support evaluation of this combination in clinical trials. Finally, the rapid loss of resistance in the absence of venetoclax may also inform dosing schedules in such trials.

## MATERIALS AND METHODS

### Cell lines, reagents and treatment

WSU-FSCCL cells were isolated from blood of a patient with leukemic phase FL and are EBV negative and *t(14;18)* positive. FC-TxFL2 cells were isolated from pleural fluid of a patient with transformed FL and they are also EBV negative and *t(14;18)* positive. All antibodies, except anti-NOXA (Thermo Scientific, Waltham, MA) were obtained from Cell Signaling Technology (Danvers, MA). Venetoclax was obtained from AbbVie Inc. (North Chicago, IL). BKM120 was provided by Novartis Pharmaceuticals Inc. (Cambridge, MA). SC772984 was purchased from Selleckchem (Houston, TX) and SP600125 was purchased from Cell Signaling Technology.

Primary lymphoma samples were obtained according to protocols approved by the Institutional Review Board of the Cleveland Clinic. Isolated primary cells were re-suspended to 2–5 × 10^6^/ml in RPMI 1640 medium containing 20% fetal bovine serum.

The parental cell lines were treated with the increasing concentrations of venetoclax 1 − 500 nM to acquired resistance. Once the resistant cell lines were established, they were sub-cultured in the presence of 500 nM venetoclax.

Dead cells were removed using Dead Cell Removal kit (Miltenyi Biotec Inc., San Diego, CA) according to manufacturer's instructions. Briefly, dead cells were labeled with magnetic MicroBeads and removed from cell suspension using positive selection column type MS placed in MACS separator.

### *In vivo* model

FC-TxFL2 cells (5 × 10^6^) were injected subcutaneously into NSG mice on an IACUC-approved protocol. Venetoclax was administered at the indicated dose once daily by gavage. In the venetoclax plus rituximab experiment, one dose of rituximab was injected in the intraperitoneal cavity 14 days after cell injection and before venetoclax treatment was initiated. Tumors were frozen or disintegrated for further experiments after the venetoclax treatment was complete or when tumors reached maximum size allowed by IACUC.

### Cytotoxicity, apoptotic assay and cell cycle analyses

Effect of drugs on cell survival was assessed with the water-soluble tetrazolium salt (WST)-1 assay. Cells were treated simultaneously with five serial dilutions of drugs for 72 H. Thereafter, the cells were incubated with WST-1 according to manufacturer's instructions (Roche, IN, USA). Cell cycle phases and the percentage of subdiploid population were determined by propidium iodide flow cytometry staining. Briefly, the cells were permeabilized with 0·05% Triton X-100 (Sigma-Aldrich, St. Louis, MO, USA)/PBS with RNase A (100 μg/ml; Fisher-Scientific, Pittsburgh, PA, USA) and stained with propidium iodide (Sigma-Aldrich). Additionally, the percentage of subdiploid cells was correlated with Annexin V-PE/7-AAD (BD Biosciences, San Jose, CA, USA) staining. The cells were stained with Annexin V-PE/7-aminoactinomycin D (7-AAD) according to the manufacturer's instructions.

### Quantitative flow cytometry assay of BCL-2 and BIM

Isolated lymphoma cells (5–10 ×10^5^ cells) were first fixed with Lyse-Fix buffer (BD558049, BD Biosciences) at 37°C for 10 min. Fixed cells were washed and re-suspended in 100 μl of FACS staining buffer (1% BSA and 2 mM EDTA in HBSS) followed by permeabilization with Perm/Wash buffer (BD557885, BD Biosciences) for 20 min at 4°C. Cells were then washed and further incubated with 20 ml of mouse IgG for 10 min at 4°C before staining with PE-BCL-2 (BD556535, BD Biosciences) or APC-BIM (supplied by AbbVie). BCL-2 and BIM expression (molecules/cell) were determined via Antibody Binding Capacity of the cells using Quantum Simply Cellular anti-Mouse IgG beads (815) and Molecules of Equivalent Soluble Fluorochrome (MESF) using Quantum APC MESF beads (823) beads (Bangs Laboratories, Fishers, IN), respectively. The standard curves were created in QuickCal^®^ software following instruction of Bangs Laboratories [10].

### Western blotting and immunoprecipitation

Whole-cell protein extracts were prepared by sonication in RIPA lysis buffer (Cell Signaling Technology) on ice. Each lane of a 4–15% Ready Tris-HCl gel (Bio-Rad, Hercules, CA, USA) was loaded with ~20 μg of protein. After electro-transfer, blots were blocked with 5% milk/TBS-T, incubated with the recommended dilutions of indicated antibodies overnight at 4°C and followed by incubation with horseradish peroxidase-conjugated anti-rabbit- or anti-mouse-secondary antibody for 1 H at room temperature.

Immunoprecipitation protocol utilizing magnetic separation was used. Briefly, cell lysates were incubated with primary antibodies overnight at 4°C. The immunocomplex solution was then transferred to a tube containing the pre-washed protein A magnetic beads and incubated for 30 minutes at room temperature. The beads were removed in the magnetic separation rack and supernatant was used for Western blotting analysis.

### Mitochondrial potential analysis

Mitochondrial membrane potential of cells was evaluated by using JC-1 (Thermo Scientific) flow cytometric staining following the manufacturer's instruction. The values of mitochondrial membrane potential were expressed as ratios of red fluorescence intensity over green fluorescence intensity.

### Statistical analysis

The values of IC_50_ and the interaction between drugs were examined according to the Chou and Talalay method [30] by using Calcusyn (Biosoft, Cambridge, United Kingdom). Statistical analysis was performed using Statistica (StatSoft, Inc., Tulsa, OK), and significance determined by employing two tailed Student test. The Cox's *F* test in the Kaplan-Meier surviving analysis for two groups was used. *P* value of < 0.05 was considered significant.

## References

[1] Gupta RK, Lister TA (1996). Current management of follicular lymphoma. Curr Opin Oncol.

[2] Tsujimoto Y, Cossman J, Jaffe E, Croce CM (1985). Involvement of the bcl-2 gene in human follicular lymphoma. Science.

[3] Chipuk JE, Moldoveanu T, Llambi F, Parsons MJ, Green DR (2010). The BCL-2 family reunion. Mol Cell.

[4] Elkholi R, Floros KV, Chipuk JE (2011). The Role of BH3-Only Proteins in Tumor Cell Development, Signaling, and Treatment. Genes Cancer.

[5] Roberts AW, Seymour JF, Brown JR, Wierda WG, Kipps TJ, Khaw SL, Carney DA, He SZ, Huang DC, Xiong H, Cui Y, Busman TA, McKeegan EM (2012). Substantial susceptibility of chronic lymphocytic leukemia to BCL2 inhibition: results of a phase I study of navitoclax in patients with relapsed or refractory disease. J Clin Oncol.

[6] Souers AJ, Leverson JD, Boghaert ER, Ackler SL, Catron ND, Chen J, Dayton BD, Ding H, Enschede SH, Fairbrother WJ, Huang DC, Hymowitz SG, Jin S (2013). ABT-199, a potent and selective BCL-2 inhibitor, achieves antitumor activity while sparing platelets. Nat Med.

[7] Roberts AW, Davids MS, Pagel JM, Kahl BS, Puvvada SD, Gerecitano JF, Kipps TJ, Anderson MA, Brown JR, Gressick L, Wong S, Dunbar M, Zhu M (2015). Targeting BCL2 with Venetoclax in Relapsed Chronic Lymphocytic Leukemia. N Engl J Med.

[8] Mohammad RM, Mohamed AN, Smith MR, Jawadi NS, al-Katib A (1993). A unique EBV-negative low-grade lymphoma line (WSU-FSCCL) exhibiting both t(14;18) and t(8;11). Cancer Genet Cytogenet.

[9] Smith MR, Jin F, Joshi I (2014). Milatuzumab and veltuzumab induce apoptosis through JNK signalling in an NF-kappaB dependent human transformed follicular lymphoma cell line. Br J Haematol.

[10] Smith ML, Chyla B, McKeegan E, Tahir SK (2016). Development of a flow cytometric method for quantification of BCL-2 family members in chronic lymphocytic leukemia and correlation with sensitivity to BCL-2 family inhibitors. Cytometry B Clin Cytom.

[11] Bjorkoy G, Lamark T, Brech A, Outzen H, Perander M, Overvatn A, Stenmark H, Johansen T (2005). p62/SQSTM1 forms protein aggregates degraded by autophagy and has a protective effect on huntingtin-induced cell death. J Cell Biol.

[12] Dai H, Ding H, Meng XW, Lee SH, Schneider PA, Kaufmann SH (2013). Contribution of Bcl-2 phosphorylation to Bak binding and drug resistance. Cancer Res.

[13] Deng X, Xiao L, Lang W, Gao F, Ruvolo P, May WS (2001). Novel role for JNK as a stress-activated Bcl2 kinase. J Biol Chem.

[14] Smith MR, Jin F, Joshi I (2004). Enhanced efficacy of therapy with antisense BCL-2 oligonucleotides plus anti-CD20 monoclonal antibody in scid mouse/human lymphoma xenografts. Mol Cancer Ther.

[15] Pro B, Leber B, Smith M, Fayad L, Romaguera J, Hagemeister F, Rodriguez A, McLaughlin P, Samaniego F, Zwiebel J, Lopez A, Kwak L, Younes A (2008). Phase II multicenter study of oblimersen sodium, a Bcl-2 antisense oligonucleotide, in combination with rituximab in patients with recurrent B-cell non-Hodgkin lymphoma. Br J Haematol.

[16] Bonavida B (2007). Rituximab-induced inhibition of antiapoptotic cell survival pathways: implications in chemo/immunoresistance, rituximab unresponsiveness, prognostic and novel therapeutic interventions. Oncogene.

[17] Davids MS, Seymour JF, Gerecitano JF, Kahl BS, Pagel JM, Roberts AW (2013). The Single-Agent Bcl-2 Inhibitor ABT-199 (GDC-0199) In Patients With Relapsed/Refractory (R/R) Non-Hodgkin Lymphoma (NHL): Responses Observed In All Mantle Cell Lymphoma (MCL) Patients. Blood.

[18] Del GM, Brown JR, Certo M, Love TM, Novina CD, Letai A (2007). Chronic lymphocytic leukemia requires BCL2 to sequester prodeath BIM, explaining sensitivity to BCL2 antagonist ABT-737. J Clin Invest.

[19] Khaw SL, Merino D, Anderson MA, Glaser SP, Bouillet P, Roberts AW, Huang DC (2014). Both leukaemic and normal peripheral B lymphoid cells are highly sensitive to the selective pharmacological inhibition of prosurvival Bcl-2 with ABT-199. Leukemia.

[20] Luciano F, Jacquel A, Colosetti P, Herrant M, Cagnol S, Pages G, Auberger P (2003). Phosphorylation of Bim-EL by Erk1/2 on serine 69 promotes its degradation via the proteasome pathway and regulates its proapoptotic function. Oncogene.

[21] Zhang W, Konopleva M, Ruvolo VR, McQueen T, Evans RL, Bornmann WG, McCubrey J, Cortes J, Andreeff M (2008). Sorafenib induces apoptosis of AML cells via Bim-mediated activation of the intrinsic apoptotic pathway. Leukemia.

[22] Bennett BL, Sasaki DT, Murray BW, O'Leary EC, Sakata ST, Xu W, Leisten JC, Motiwala A, Pierce S, Satoh Y, Bhagwat SS, Manning AM, Anderson DW (2001). SP600125, an anthrapyrazolone inhibitor of Jun N-terminal kinase. Proc Natl Acad Sci USA.

[23] Choudhary GS, Al-Harbi S, Mazumder S, Hill BT, Smith MR, Bodo J, Hsi ED, Almasan A (2015). MCL-1 and BCL-xL-dependent resistance to the BCL-2 inhibitor ABT-199 can be overcome by preventing PI3K/AKT/mTOR activation in lymphoid malignancies. Cell Death Dis.

[24] Rosich L, Saborit-Villarroya I, Lopez-Guerra M, Xargay-Torrent S, Montraveta A, Aymerich M, Villamor N, Campo E, Perez-Galan P, Roue G, Colomer D (2013). The phosphatidylinositol-3-kinase inhibitor NVP-BKM120 overcomes resistance signals derived from microenvironment by regulating the Akt/FoxO3a/Bim axis in chronic lymphocytic leukemia cells. Haematologica.

[25] Kim KW, Moretti L, Mitchell LR, Jung DK, Lu B (2009). Combined Bcl-2/mammalian target of rapamycin inhibition leads to enhanced radiosensitization via induction of apoptosis and autophagy in non-small cell lung tumor xenograft model. Clin Cancer Res.

[26] Malik SA, Orhon I, Morselli E, Criollo A, Shen S, Marino G, BenYounes A, Benit P, Rustin P, Maiuri MC, Kroemer G (2011). BH3 mimetics activate multiple pro-autophagic pathways. Oncogene.

[27] Saleem A, Dvorzhinski D, Santanam U, Mathew R, Bray K, Stein M, White E, DiPaola RS (2012). Effect of dual inhibition of apoptosis and autophagy in prostate cancer. Prostate.

[28] Gu TL, Tothova Z, Scheijen B, Griffin JD, Gilliland DG, Sternberg DW (2004). NPM-ALK fusion kinase of anaplastic large-cell lymphoma regulates survival and proliferative signaling through modulation of FOXO3a. Blood.

[29] Lee JS, Tang SS, Ortiz V, Vo TT, Fruman DA (2015). MCL-1-independent mechanisms of synergy between dual PI3K/mTOR and BCL-2 inhibition in diffuse large B cell lymphoma. Oncotarget.

[30] Chou TC, Talalay P (1984). Quantitative analysis of dose-effect relationships: the combined effects of multiple drugs or enzyme inhibitors. Adv Enzyme Regul.

